# A forced convection of water aluminum oxide nanofluid flow and heat transfer study for a three dimensional annular with inner rotated cylinder

**DOI:** 10.1038/s41598-022-21004-x

**Published:** 2022-10-06

**Authors:** Usman, Abid Ali Memon, Metib Alghamdi, Taseer Muhammad

**Affiliations:** 1 Department of Computer Science, National University of Sciences and Technology, Balochistan Campus (NBC), Quetta, 87300 Pakistan; 2 Department of Mathematics and Social Sciences, Sukkur IBA University, Sukkur Sindh, 65200 Pakistan; 3 Department of Mathematics, College of Sciences, King Khalid University, Abha, 61413 Saudi Arabia

**Keywords:** Applied mathematics, Computational science

## Abstract

The article examines a water alumina nanofluid and heat transfer through the three-dimensional annular. The annular is constructed by the two concentric cylinders in which the inner cylinder can rotate along the tangential direction at a constant speed. A slip boundary condition will be imposed to vanish the viscous effect in the vicinity of the outer cylinder wall. Moreover, the rotating cylinder is kept at a hot temperature, and the outer one is at a cold temperature. A three-dimensional incompressible Navier Stokes and energy equations were carried in cylindrical coordinates. The simulation was observed using the emerging computational tool of COMSOL Multiphysics 5.6, which implements Least Square Galerkin's scheme of finite element method. The parametric study will be done by altering the speed of rotation of the inner cylinder from 1 to 4, volume fraction from 0.001 to 0.9, and the aspect ratio from 0.4 to 0.6 for a fixed Reynolds number of 35,000. The results will be displayed with graphs and tables for average values of the Nusselt number, the percentage change in the temperature, and the skin friction at the middle plan. It was found that the average Nusselt number at the middle of the annular increases before the volume fraction of 0.2 and then decreases for all values of the volume fraction for a fixed rotation of the inner cylinder. The average percentage change relative to the inner cylinder's hot temperature decreases with the volume fraction increase for the fixed rotation. Also, it was found that the quantity of nanoparticles in the domain is improving the average skin friction in the middle of the channel, and it can be reduced by improving the rotation of the inner cylinder by about 10–23% strictly depending upon the aspect ratio for a particular case.

## Introduction

For decades, fluid flow and heat transfer through the three-dimensional annular channel were largely studied. They are investigated not only numerically but also experimentally. They are important because they can be used in the practical applications of heat exchangers, thermal storage systems, and solar collector cells^1–3^. Due to global development over the last decade, the energy demand has enlarged. Innumerable techniques have been developed to save energy as well as cost. These techniques especially involve changing the configuration of the shapes of the channel and materials like nanofluid, which retains a high rate of thermal conductivity. In engineering applications, annular geometry is widely studied with different configurations of the constructions. The inner cylinders of the annular geometry can be set as square, triangular, rhombic, conical, rectangular, and circular for the fluid flow and heat transfer study^4–7^.

Initially, this geometry's importance or value in heat transfer rate was explained in^8–13^, where this geometry was studied for mixed, natural, and forced convection. Later, the investigation of heat transfer through the annular was studied by^14–17^. These numerical studies were based on the suspension of water aluminum oxide through the horizontal annular channel to enhance the heat transfer efficiency through mixed convection. After that^18,19^ investigated the annular channel for thermal transfer with laminar as well as turbulent flow by setting the inner cylinder in an elliptical shape. It was suggested that nanofluids like Al2O3, ZnO, CuO, and SiO2 are good in increasing the heat transfer rate. It was deducted that lower values of the Nusselt number were found using only base fluid. After implementing the first and second law of thermodynamics, the three-dimensional annular channel was again investigated with water-alumina nanofluid for hydraulic and thermal properties^20^.

An incredible rate increase was found in thermal enrichment with the addition of the nanoparticles. Also, a major shortcoming was stated regarding friction coefficient and entropy generation. The heat transfer rate was calculated through a horizontal annular channel by considering the water-alumina and water-copper oxide^21,22^. It was found that the convection process dominated the conduction process when $Ra \ge 10^{4}$ a direct relationship was seen between the nanoparticle volume fraction and the average Nusselt number. Later, the annular with the porous medium was investigated^23,24^ for natural convection with the influence of nanofluid transport. It was found that the energy transformation declines with the increase of volume fraction in the case of aluminum oxide without the addition of the porosity in the tube. A theoretical investigation was done through the annular in space coordinates^25^, in which the optimum particle filling was determined to get the maximum heat transfer rate for different types of fluids. He explained that a certain critical value of the volume fraction occurs for which the viscosity of the nanomaterial has excessive growth. It was also explained that volume fraction and the average temperature are increased by decreasing nanoparticle size. Such impact of maximum particle filling was seen in the articles^26–28^. An enclosure with a moving barrier was also observed for the nanofluid transport due to the impact of the Lorentz forces under the magnetic field effect^29^. It was indicated that the Lorentz forces could be the reason for reducing the strength of the magnetic field and direction. In observation of the laminar nanofluid flow through the lid-driven cavity under the partial slip condition^30^, it was found that there exist some critical values or the partial slip parameter, for which the convection is decreasing. A magnetohydrodynamics mixed convection problem was simulated for transporting a two-sided lid-driven cavity filled with porous material under the partial slip parameters^31^. It was found that the heat transfer rate directly correlates with the volume fraction of nanoparticles for moving walls in either direction. Also, more material can be found for the transportation of the nanofluid through the different shapes with different approaches, either by magnetic effect or entropy generation^32^. Also, more advantages can be taken to increase the heat transfer rate from the transportation of the nanofluid when observed by the analytical, numerical, and experimental approach, or sometimes it can be given a better output when using the combination of the nanoparticles. It can be made more beneficiary by adding chemical engineering, magnetic, or electric field, for some relevant articles are^33–37^.

After investigating the literature and the importance of nanofluids in enhancing the heat transfer rate, this article will investigate the three-dimensional annular that is settled vertically. The observed channel is constructed using the two concentric cylinders where the inner cylinder rotates along the tangential direction. Due to ignoring the viscous effect at the surface of the outer cylinder, slip boundary conditions are applied to it. A hot and cold temperature was imposed on the inner and outer concentric cylinder. The water and aluminum oxide mixture is allowed to enter from the top of the channel in the volume fractions from 0.001 to 0.9. The numerical investigation will be done using the finite element method package COMSOL Multiphysics 5.6. The parametric study will be done using the rotation parameter of the inner cylinder from 1 to 4 and the aspect ratio from the inner to the outer cylinder from 0.4 to 0.6. For thermal conductivity and viscosity of nanofluid^38^ and^39^ models will be employed, respectively. The results will be validated by using the mesh independent study and matching the results with the available correlations^40–43^. The key variables like the average Nusselt number, the average change in the temperature to the hot temperature of the inner cylinder, and the average skin friction will be discussed by 2 of the three parameters in the middle of the channel. All average values and their post-processing will be explained in the tables.

## Problem formulation and governing equations

**Figure 1:**
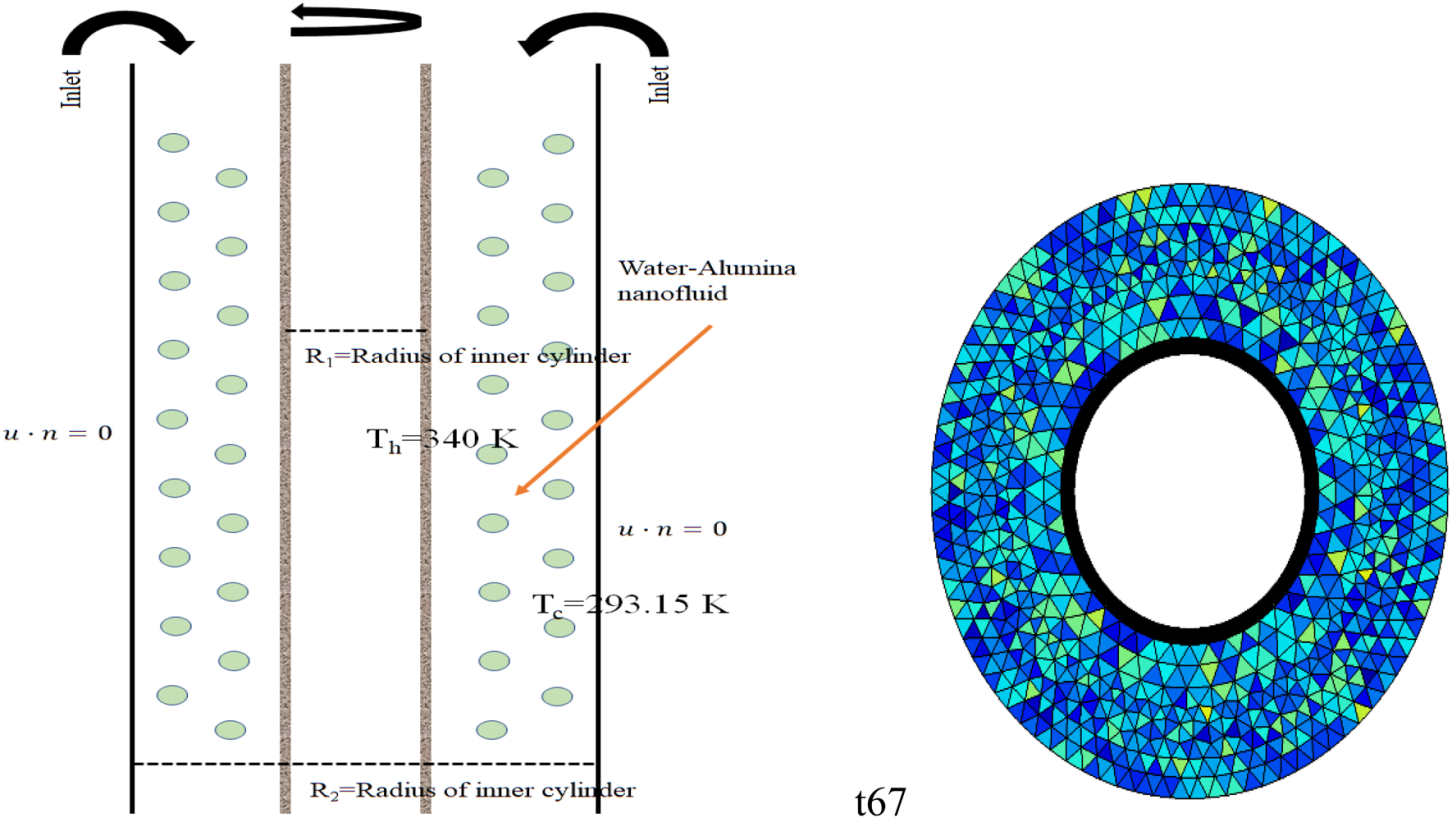
(**a**) Schematic diagram of the channel (**b**) Front view of the channel.

Figure 1a and b shows the schematic diagram of the channel chosen to observe the water alumina nanofluid in a three-dimensional coordinate system. The channel consists of two concentric cylinders in which the outer cylinder faces the slip boundary condition for the sack to ignore the viscous effect at the vicinity of the boundary and also faces a cooling temperature of T_c_ = 293.15. The inner cylinder is not fixed and capable of rotating with a constant speed $\omega$ and facing a hot temperature of T_h_ = 340 K. Let R_1_ and R_2_ be the radius of the inner and outer cylinder, respectively, then the aspect ratio is defined to be Ar = R_1_/R_2_ and which will be varying from 0.4 to 0.6 for the current problem. The total length L of the channel is taken twice of router R_2_. The rotation of the inner cylinder will be tested for the speed of 1–4 m/s along the y-axis, and the volume fraction of the nanofluid $\phi$ will be tested for 0.001, 0.01, 0.1, and 0.9. The three-dimensional annular is settled vertically. The inlet velocity u_in_ in the z-direction was imposed from the top of the channel as shown in Fig. 1a, calculated with Re = 35,000. The thermophysical properties of the water alumina are described in Table 1

**Table 1:** Thermo-physical properties and other parameters for computer simulations ^38,39^.

Property Name	Value/Range	Description
$\rho_{f}$	997.1 [kg/m^3^]	Density of water
$\rho_{s}$	3970 [kg/m^3^]	The density of the Aluminum oxide
$(c_{p} )_{f}$	4179 [J/(mol K)]	Heat capacity of water at constant pressure
$(c_{p} )_{s}$	765 [J/(mol K)]	Heat capacity of Aluminum oxide
$\kappa_{f}$	0.613 [W/(m K)]	Thermal conductivity of water
$\kappa_{s}$	40 [W/(m K)]	Thermal conductivity of alumina
$\beta_{f}$	21E-5 [1/K]	Thermal expansion of water
$\beta_{s}$	0.85E-5 [1/K]	Thermal expansion of water
$\phi$	0.001, 0.01, 0.1, 0.2	Volume fraction
$\rho_{nf}$	$(1 - \phi )\rho_{f} + \phi \rho_{s}$	Density of nanofluid
$\mu_{f}$	0.00081 [Pa*s]	Viscosity of water
$\mu_{nf}$	$\mu_{f} (1 + 7.3\phi + 123\phi^{2} )$	Dynamic viscosity of nanofluid
g	9.8 m/s^2^	Acceleration due gravity
$\beta_{nf}$	$(1 - \phi )\beta_{f} + \phi \beta_{s}$	Thermal expansion of the nanofluids
$\kappa_{nf}$	$\kappa_{f} \frac{{\kappa_{s} + 2\kappa_{f} - 2\phi (\kappa_{f} - \kappa_{s} )}}{{\kappa_{s} + 2\kappa_{f} + \phi (\kappa_{f} - \kappa_{s} )}}$	Thermal conductivity of nanofluid Maxwell Garnet model
$(\rho c_{p} )_{f}$	$\rho_{f} (c_{p} )_{f}$	Heat capacitance of fluid
$(\rho c_{p} )_{s}$	$\rho_{s} (c_{p} )_{s}$	Heat capacitance of solid
$(\rho c_{p} )_{nf}$	$(1 - \phi )(\rho c_{p} )_{f} + \phi (\rho c_{p} )_{s}$	Heat capacitance of nanofluid
$\alpha_{nf}$	$\frac{{\kappa_{nf} }}{{(\rho c_{p} )_{nf} }}$	Thermal diffusivity of nanofluid
$T_{ref}$	293.15 [K]	Reference temperature of the square cavity
$D_{h}$	4Area of the channel/Perimeter	Hydraulic diameter
$u_{in}$	$\frac{{Re\mu_{nf} }}{{D_{h} \rho_{nf} }}$	Inlet velocity
Re	35,000	Reynolds number
Ar	0.4, 0.5, 0.6	Radius of cylinder
$\mathop m\limits^{ \bullet }$	–	Mass flow rate
$\mathop V\limits^{ \to } = < v_{r} ,v_{\theta } ,v_{z} >$	–	Velocity field with its radial, tangential and axial components, respectively

A water and aluminum oxide nanofluid investigate the fluid flow and heat transfer problem. The nanofluid with variable physical properties used in the problem is assumed to be dilute, continuous, and Newtonian. The heat conduction and energy equation also assumes that the energy loss due to compression, dispersion of the particles, and viscous dissipation are negligible. Moreover, it was considered that the base fluid water and the nanoparticles alumina would remain in thermal equilibrium, and no heat source, chemical reaction, radiation process, and external force would be involved. We can express the governing Navier Stokes equations and energy equation^44,45^ and the boundary condition^46^ as follow:

Continuity equation:

1 $$ \frac{1}{r}\frac{\partial }{\partial }(ru_{r} ) + \frac{1}{r}\frac{{\partial u_{\theta } }}{\partial \theta } + \frac{{\partial u_{z} }}{\partial z} = 0 $$

Radial Component

2 $$ \rho_{nf} \left( {u_{r} \frac{{\partial u_{r} }}{\partial r} + \frac{{u_{\theta } }}{r}\frac{{\partial u_{r} }}{\partial \theta } + u_{z} \frac{{\partial u_{r} }}{\partial z} - \frac{{u_{\theta }^{2} }}{r}} \right) = - \frac{\partial p}{{\partial r}} + \mu_{nf} \left[ {\frac{1}{r}\frac{\partial }{\partial r}\left( {r\frac{{\partial u_{r} }}{\partial r}} \right) + \frac{1}{{r^{2} }}\frac{{\partial^{2} u_{r} }}{{\partial \theta^{2} }} + \frac{{\partial^{2} u_{r} }}{{\partial z^{2} }} - \frac{{u_{r} }}{{r^{2} }} - \frac{2}{{r^{2} }}\frac{{\partial u_{\theta } }}{\partial \theta }} \right] $$

Tangential component

3 $$ \rho_{nf} \left( {u_{r} \frac{{\partial u_{\theta } }}{\partial r} + \frac{{u_{\theta } }}{r}\frac{{\partial u_{\theta } }}{\partial \theta } + u_{z} \frac{{\partial u_{\theta } }}{\partial z} - \frac{{u_{r} u_{\theta } }}{r}} \right) = - \frac{1}{r}\frac{\partial p}{{\partial \theta }} + \mu_{nf} \left[ {\frac{1}{r}\frac{\partial }{\partial r}\left( {r\frac{{\partial u_{\theta } }}{\partial r}} \right) + \frac{1}{{r^{2} }}\frac{{\partial^{2} u_{\theta } }}{{\partial \theta^{2} }} + \frac{{\partial^{2} u_{\theta } }}{{\partial z^{2} }} - \frac{{u_{\theta } }}{{r^{2} }} + \frac{2}{{r^{2} }}\frac{{\partial u_{r} }}{\partial \theta }} \right] $$

Axial component

4 $$ \rho_{nf} \left( {u_{r} \frac{{\partial u_{z} }}{\partial r} + \frac{{u_{\theta } }}{r}\frac{{\partial u_{z} }}{\partial \theta } + u_{z} \frac{{\partial u_{\theta } }}{\partial z}} \right) = - \frac{1}{r}\frac{\partial p}{{\partial z}} + \mu_{nf} \left[ {\frac{1}{r}\frac{\partial }{\partial r}\left( {r\frac{{\partial u_{z} }}{\partial r}} \right) + \frac{1}{{r^{2} }}\frac{{\partial^{2} u_{z} }}{{\partial \theta^{2} }} + \frac{{\partial^{2} u_{z} }}{{\partial z^{2} }}} \right] $$

Energy equation

5 $$ \left( {\rho c_{p} } \right)_{nf} \left( {v_{r} \frac{\partial T}{{\partial r}} + v_{\theta } \frac{\partial T}{{\partial \theta }} + v_{z} \frac{\partial T}{{\partial z}}} \right) = \frac{1}{{\kappa_{nf} }}\left[ {\frac{1}{r}\frac{\partial }{\partial r}\left( {\frac{\partial T}{{\partial r}}} \right) + \frac{1}{{r^{2} }}\frac{{\partial^{2} T}}{{\partial \theta^{2} }} + \frac{{\partial^{2} T}}{{\partial z^{2} }}} \right] $$

Boundary conditions

$$ \begin{gathered} At \, z = 2L: \, v_{r} = v_{\theta } = 0,{\text{ and }}v_{z} = f({\text{Re}} ), \, \frac{\partial T}{{\partial n}} = 0 \hfill \\ At \, z = 0: \, v_{r} = v_{\theta } = v_{z} = 0, \, p_{0} = 0 \hfill \\ At \, r = R_{1} : \, T = T_{c} , \, \mathop \omega \limits^{ \to } \cdot n = 0,{\text{ Where }}\mathop \omega \limits^{ \to } = < 0,0,\omega > \hfill \\ At \, r = R_{2} : \, T = T_{h} , \, u \cdot n = 0, \, \hfill \\ \end{gathered} $$

where ${\mathbf{u}} = < v_{r} ,v_{\theta } ,v_{z} >$ is the velocity of the nanofluid, and n is the normal vector to the surface?

Computational parameters

The other computational parameters are enlisted as follows:

Heat flux Q:$Q = \mathop m\limits^{ \bullet } (c_{p} )_{nf} (T_{h} - T_{c} )$ Heat transfer coefficient: $h = \frac{Q}{{T - T_{b} }}$.

Bulk Temperature:$T_{b} = \frac{{T_{h} + T_{c} }}{2}$ Local Nusselt number along the ith direction: $Nu_{i} = \frac{h \, i}{{k_{nf} }}$.

Average Nusselt number: $Nu_{avg} = \frac{1}{A}\int_{{R_{1} }}^{{R_{2} }} {Nu(r,\theta ,z)dA}$ Prandtl Number: $\Pr = \frac{{\mu_{nf} (c_{p} )_{nf} }}{{\kappa_{nf} }}$.

Grashof Number: $Gr = \frac{{g\beta_{f} (T_{h} - T_{c} )D_{h}^{3} }}{{\eta^{2} }}$ Skin friction coefficient $S_{f} = \frac{{\tau_{w} }}{{\frac{1}{2}\rho_{nf} u_{mean}^{2} }}$.

COMSOL Procedure

**Figure 2:**
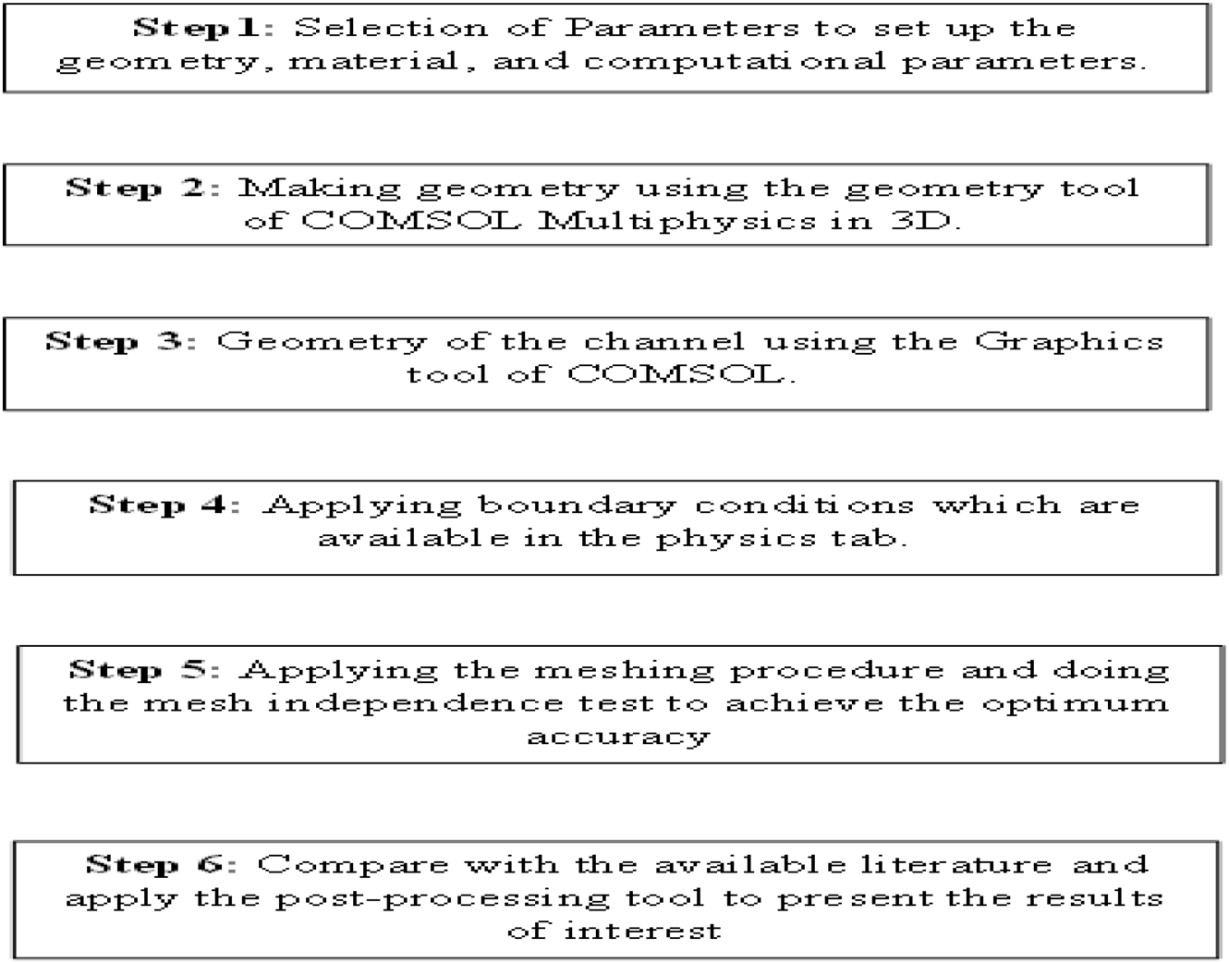
The Working Wagon Wheel of COMSOL.

**Figure 3:**
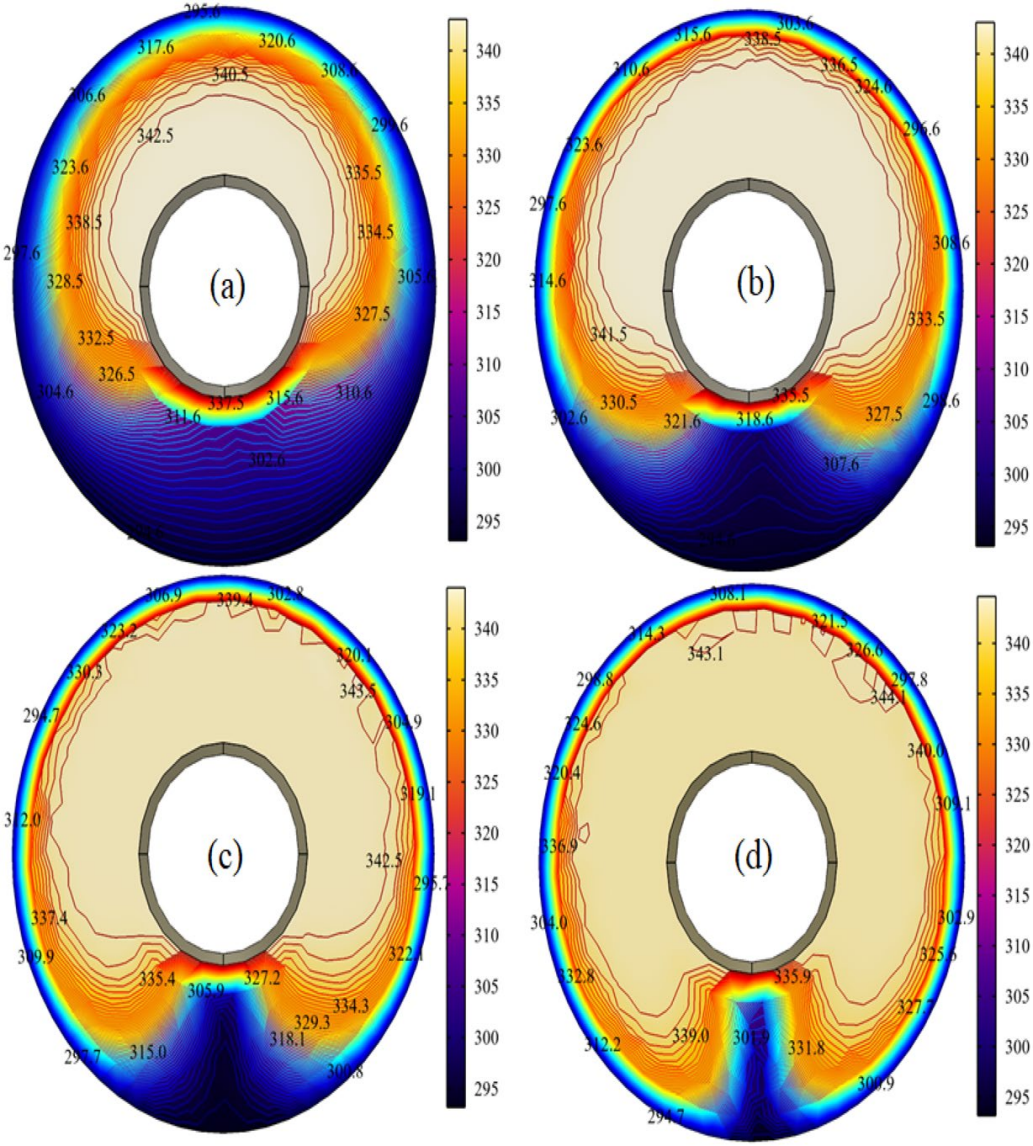
Surface and contour plots of the temperature when $\phi = 0.001$ and Ar = 0.4 at (**a**) $\omega = 1$ (**b**) $\omega = 2$ (**c**) $\omega = 3$ (**d**) $\omega = 4$.

This article will observe the dynamical behavior of water alumina nanofluid and heat transfer using the commercial tool COMSOL Multiphysics. A java code will be developed using the least square Galerkin's Scheme. The COMSOL working flow chart is expressed as follows in Fig. 2:

After successfully developing the simulation, the temperature distribution along the domain is given below in Fig. 3:

### Mesh independent test and validity

**Figure 4:**
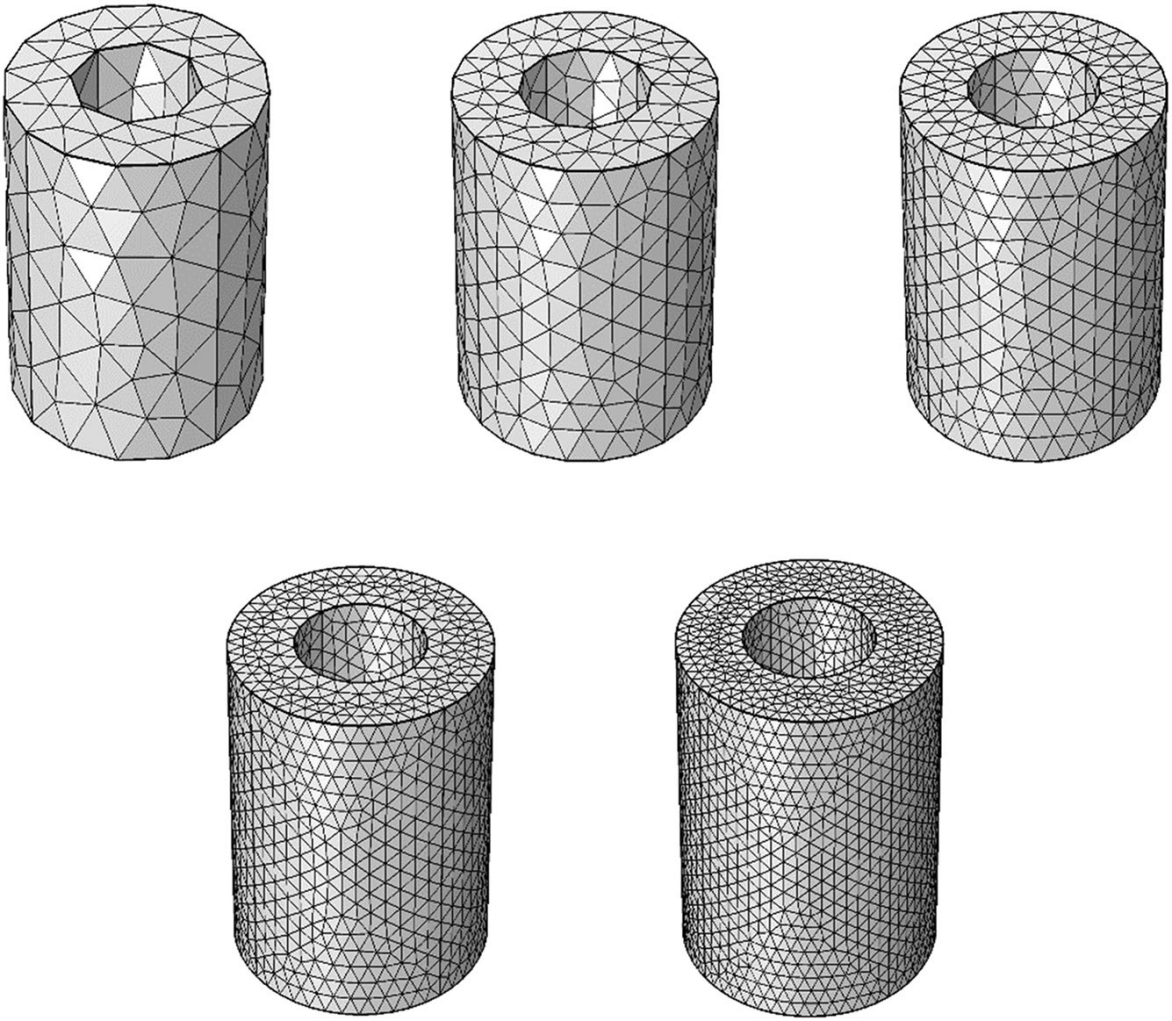
The meshing procedure of the finite element method in the 3-dimensional annulus.

**Figure 5:**
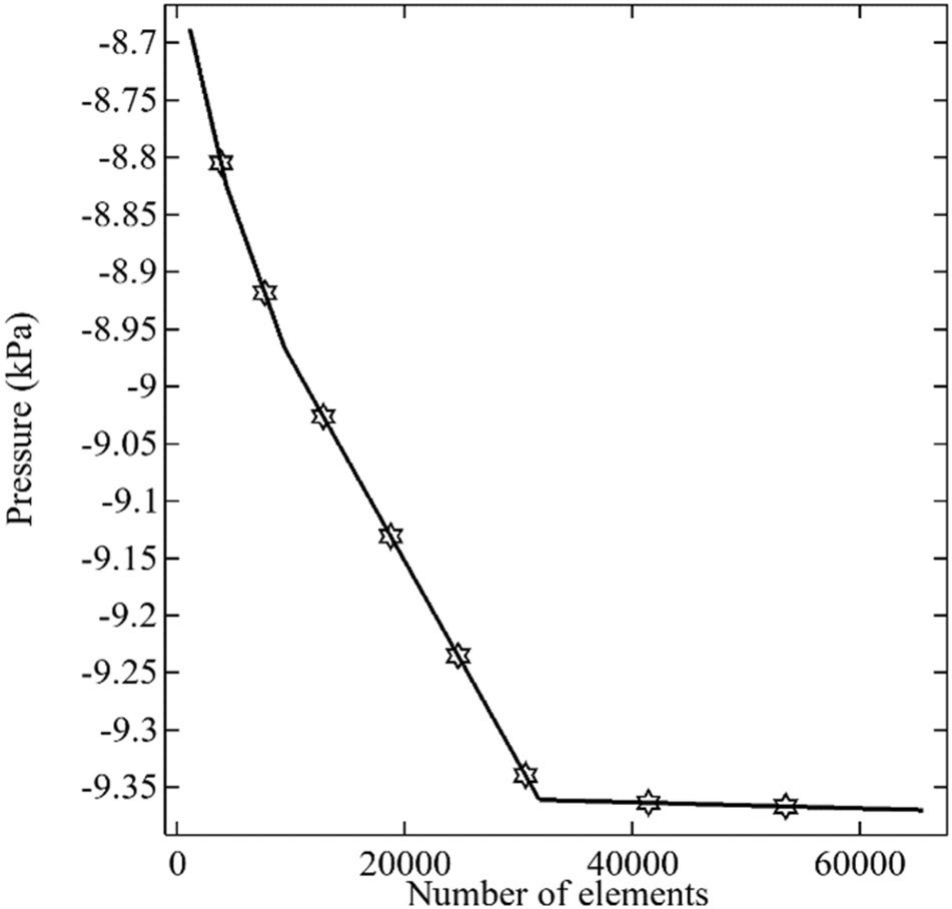
The mesh independency test for the pressure at the outlet of the domain.

The beauty of numerical methods like the finite element method is to search out the solution by decomposing the domain of interest into smaller subdomains. To decompose a domain into a smaller subdomain is called the meshing process. The smaller subdomains are called elements, and corner points are called nodes. At which the required variables will be calculated using the weighted residual procedure. The accuracy or better estimation of the solution depends upon the density of the meshing process because each better accuracy of one node will lead to better accuracy of the second node. The annular's current geometry is investigated for the water alumina nanofluid with heat transfer. The meshing process is applied to the three-dimensional geometry with the help of tetrahedral elements see Fig. 4. We obtained the numerical solution for the average pressure at the outlet for $\phi$ = 0.001, $\omega$ = 1, and Ar = 0.4 by using the different number of elements. The numerical solution for the average pressure at the outlet is getting the mesh independence for the number of elements greater than 30,000 see Fig. 5. Therefore, the current problem has been solved with a large number of elements, 64,428.

After performing a mesh independence test, we attempted to compare the average Nusselt number and the axial direction with different correlations accessible in the literature^35–38^**.** It can be detected in Table 2. The numerical results for the average Nusselt are significantly closer to the correlation^40^**.** Although, we argued that whatever we will be present in the study will have a preciseness of 90%.

**Table 2:** Comparison table of the Nusselt number.

z	Present Work	^40^ $0.023\Pr^{0.4} {\text{Re}}_{x}^{0.8}$	^41^ $0.00805\Pr^{1/3} {\text{Re}}_{x}^{0.8}$	^42^ $0.00789\Pr^{0.4} {\text{Re}}_{x}^{0.8}$	^43^ $0.012\Pr^{0.4} ({\text{Re}}_{x}^{0.87} - 280)$
1.483	171.27	199.75	62.381	68.524	210.41
1.485	171.27	199.76	62.382	68.526	210.42
1.494	171.31	199.81	62.397	68.542	210.47
1.518	171.38	199.89	62.424	68.571	210.57
1.539	171.47	199.99	62.456	68.607	210.69
1.545	171.5	200.03	62.467	68.618	210.73
1.552	171.53	200.06	62.477	68.63	210.77
1.580	171.65	200.2	62.52	68.677	210.94
1.590	171.69	200.25	62.537	68.695	211
1.604	171.72	200.28	62.546	68.705	211.03
1.604	171.72	200.28	62.546	68.706	211.04
1.605	171.72	200.29	62.547	68.707	211.04
1.616	171.74	200.31	62.554	68.714	211.06
1.617	171.74	200.31	62.555	68.715	211.07
1.621	171.78	200.36	62.569	68.731	211.12
1.640	171.94	200.54	62.626	68.793	211.34
1.651	172.01	200.63	62.654	68.824	211.44
1.658	172.06	200.68	62.669	68.841	211.5
1.666	172.11	200.74	62.69	68.864	211.58

## Result discussion

### Average Nusselt number on the plane in the middle

**Figure 6:**
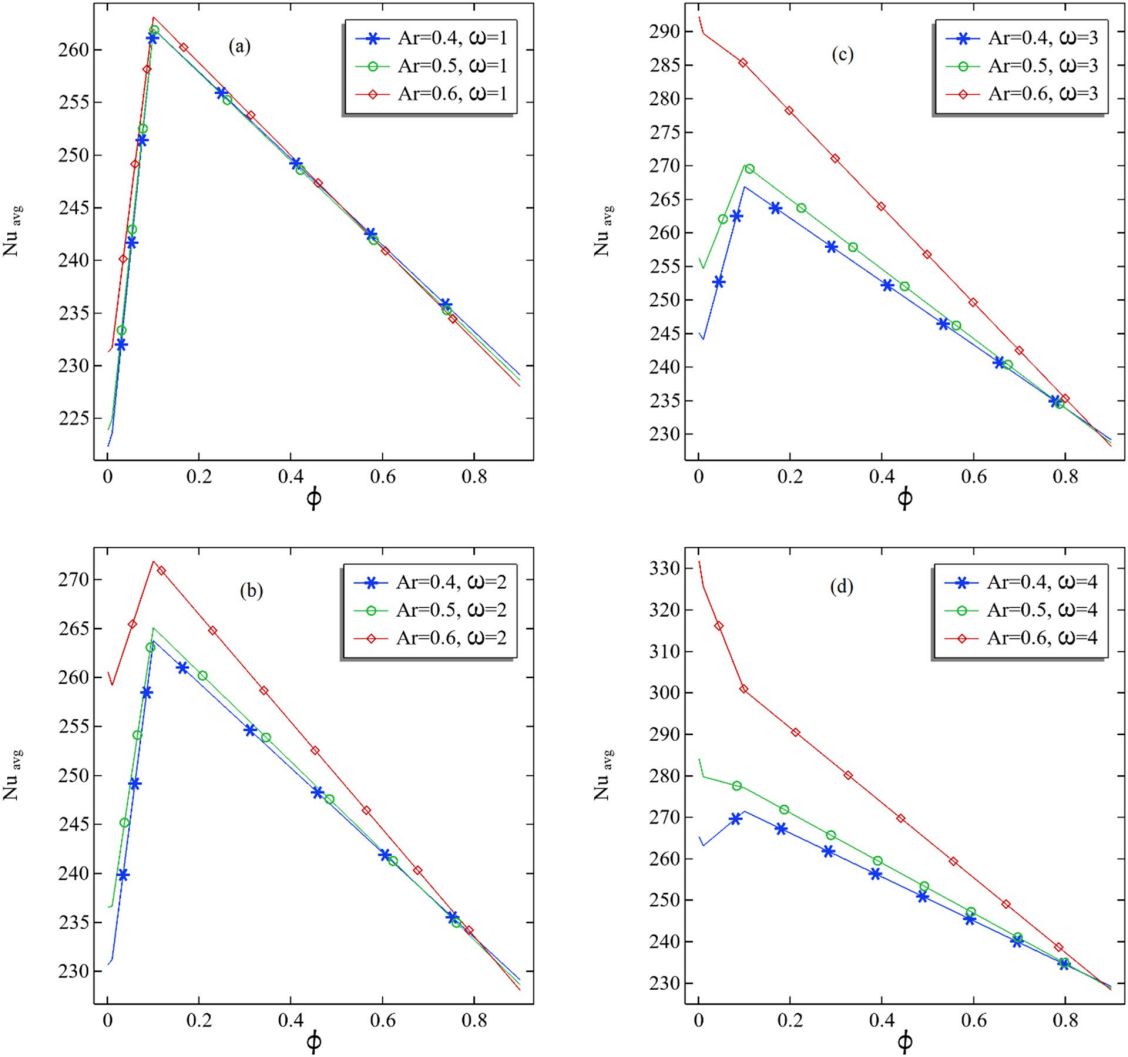
(**a**–**d**) The average Nusselt number at the middle plane with increasing volume fraction of the nanofluids.

A water alumina nanofluid and heat transfer have been observed for the three-dimensional annular in which the lower cylinder is rotated in an anticlockwise direction with speed $\omega$. To measure the convection and conduction progress in the tube, it is traditional to measure the Nusselt number. We choose the middle plane z = 1 to measure the average Nusselt number. In Fig. 6a–d, the measure of the average Nusselt number is presented by fixing the rotational speed of the inner cylinder, and the pattern is checked by increasing the volume fraction $\phi$. In Fig. 6a, we can see that the average Nusselt number increases for the volume fraction before 0.2 and decreases with the increasing volume fraction for the rotational speed of $\omega$ = 1. It can also be understood that increasing the aspect ratio from the average Nusselt boosts a little. Moreover, in Fig. 6b, we found that the average Nusselt number is also improving with increasing volume fraction as well as the aspect ratio for a fixed rotational speed of the cylinder. The increment in Nusselt number before 0.2 is found can be deducted in the case Fig. 6a–c with $\omega$ = 1–3, but look at the case in Fig. 6d for the rotational speed of $\omega$ = 4, the average Nusselt number is very quickly decreasing in the aspect ratio 0.6 with increasing volume fraction. It also can be concluded that to keep better conduction than convection, it is essential to keep the aspect ratio very high and the rotational speed of the lower cylinder. To observe the convection better than the conduction, keep the volume fraction lower than 0.2 with the moderate rotational speed of the inner cylinder.

**Figure 7:**
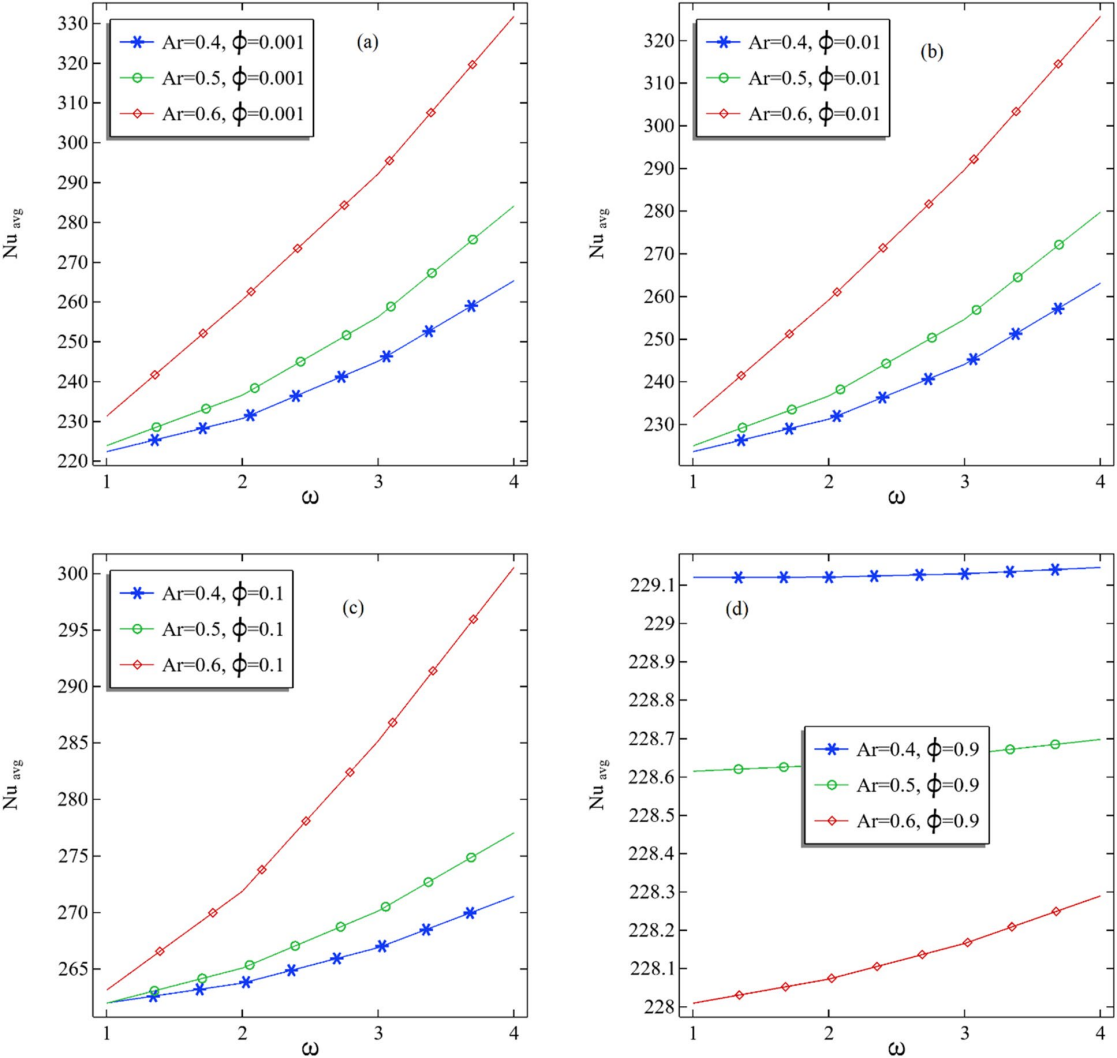
(**a**–**d**) The average Nusselt number at the middle plane with the increasing rotation rate of the inner cylinder.

In Fig. 7a–d, the graphs are produced to check the pattern of the average Nusselt number by increasing the rotational speed of the inner cylinder for each volume fraction. In Fig. 7a, it can be seen that the average Nusselt number at the midplane is increasing with an increase in rotational speed for all fixed ratios. It can be deduced that the average Nusselt for the large aspect ratio is increasing very fast and reaches up to 330 for the speed of $\omega$ = 4 at $\phi$ = 0.001. It can be perceived that with increasing the rotational speed of the inner cylinder, the relationship between the average Nusselt number and the rotational speed was found linear for constant volume fractions. The larger the aspect ratio, the larger the average Nusselt number, but the difference increases by increasing the nanofluid volume compared to the cases of Fig. 7b–d. Although, an obvious impact of volume fraction with increasing aspect ratio can be seen on the average Nusselt number at the middle plane of the annular.

Table 3 presents the numerical results for the average Nusselt number with all parameters used in the table. Also, we calculated the percentage increase or decrease in the average Nusselt number. From Table 3, it can be seen that when switching from aspect ratio 0.4 to 0.5, the most significant increment in average Nusselt can be determined in the case when $\phi$ = 0.001 and $\omega$ = 4, which is about 7.081, whereas the maximum decrement can be observed for the case when $\phi$ = 0.9 and $\omega$ = 1. Similarly, when the aspect ratio changes from 0.5 to 0.6, the most significant increment can be found when $\phi$ = 0.001 with $\omega$ = 4. Also, the most considerable decrement can be seen in Table 3 when $\phi$ = 0.9 with $\omega$ = 1. Therefore, if the volume fraction is larger but not enough, increasing the inner cylinder's rotational speed will improve the convection process in the three-dimensional annular.

**Table 3:** The details of the average number at the middle plane of the annular tube with percentage increase when switching from one aspect ratio to near one.

$\phi$	$\omega$	Ar = 0.4	Ar = 0.5	% Change from Ar = 0.4 to Ar = 0.5	Ar = 0.6	% Change from Ar = 0.5 to Ar = 0.6
0.001	1	222.3397	223.8546	0.681335	231.2771	3.315797
0.001	2	230.6709	236.5478	2.54775	260.5478	10.14596
0.001	3	245.0698	256.2049	4.543645	292.1803	14.04167
0.001	4	265.2793	284.0639	7.081059	331.7192	16.77628
0.01	1	223.5791	224.9442	0.610583	231.7105	3.007958
0.01	2	231.203	236.6408	2.351961	259.1605	9.516408
0.01	3	244.0615	254.6264	4.328779	289.7031	13.77578
0.01	4	263.0583	279.7331	6.338834	325.6172	16.40281
0.1	1	261.9765	261.9556	− 0.00797	263.1202	0.444555
0.1	2	263.7381	265.0702	0.505095	271.867	2.564163
0.1	3	266.872	270.1128	1.214348	285.1623	5.571566
0.1	4	271.4099	277.0334	2.071971	300.5197	8.477771
0.9	1	229.1195	228.6143	− 0.22047	228.0095	− 0.26458
0.9	2	229.1207	228.6307	− 0.21385	228.0723	− 0.24421
0.9	3	229.1293	228.6584	− 0.20552	228.1652	− 0.2157
0.9	4	229.1459	228.6973	− 0.19574	228.2892	− 0.17848

### Average percentage change in the temperature at the middle plane of the annular

**Figure 8:**
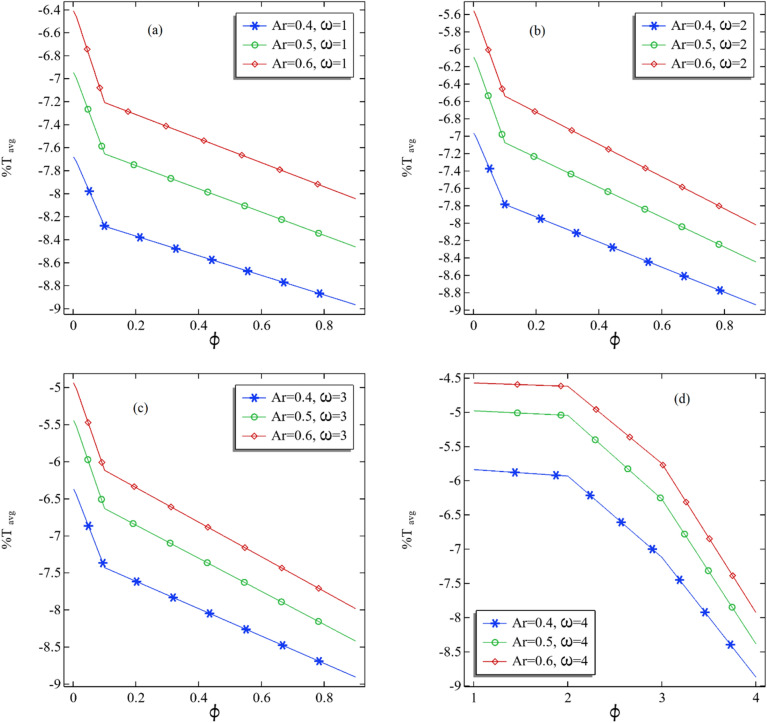
(**a**–**d**) The average percentage change %T at the middle plane of the annular with the increasing volume fraction of the nanofluid.

In this portion, we intend to illustrate the average percentage change (increase or decrease) in the temperature by assessing the hot temperature as the initial temperature assigned to the inner rotational cylinder. The average temperature change is described by fixing the volume fraction parameters and the inner cylinder's rotational speed. In Fig. 8a–d, the average percentage change in the temperature is depicted by changing the volume fraction with a fixed rotation of the inner cylinder in the middle of the channel. For a fixed rotation of the inner cylinder, the average percentage change in the temperature decreases by increasing the volume fraction see Fig. 8a–d. The average percentage change in the temperature decreases significantly by reducing the aspect ratio. This decrement in the percentage change in the temperature defines that the temperature relative to hot temperature at the inner cylinder decreases with the increase in volume fraction. Also, by increasing the distance between two cylinders, the decrement in the average percentage is improved further. However, a quick decrease in the average percentage can be seen for all the cases before the volume fraction of 0.2. In all the cases, the maximum average percentage change can be seen at the volume fraction near zero, i.e., 0.001.

**Figure 9:**
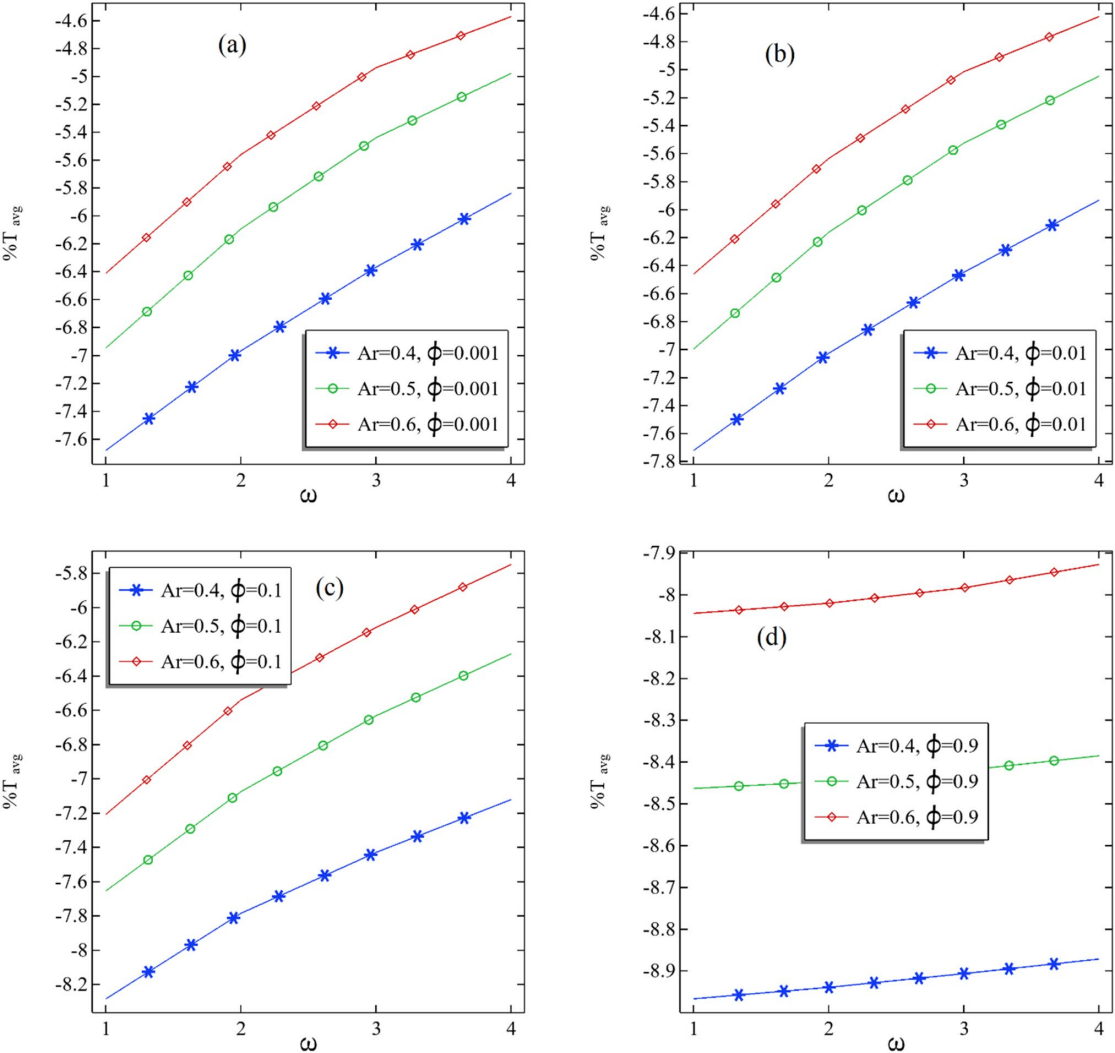
(**a**–**d**) The average percentage change %T at the middle plane of the annular with the increasing volume fraction of the nanofluid.

In Fig. 9a–d, the average percentage change in the temperature relative to hot temperature is perceived by changing the rotation and fixing the volume fraction. It can be discerned that the average percentage change increases with an increase in the rotation of the inner cylinder. Moreover, the average percentage increment is improving by enhancing the aspect ratio, which comprises Fig. 9b and c. For the current problem, the maximum and average percentage modification can be seen for the case $\omega$ = 4, further strengthened by an increase in the volume fraction.

In Table 4, the all-average percentage change in the temperature is expressed with the difference when we switch from one aspect ratio to another. These positive differences show that the average percentage of increment in the temperature increases when switching from one aspect ratio to another. The maximum and average percentage difference can be seen in the case when $\phi$ = 0.001 and $\omega$ = 3 when the aspect ratio between the cylinder is switched from 0.4 to 0.5. When switched from 0.5 to 0.6 in the aspect ratio, the maximum average percentage can be seen in cases $\phi$ = 0.01 and $\omega$ = 3.

**Table 4:** The average percentage change for all the parameters used in the problem.

$\phi$	$\omega$	Ar = 0.4	Ar = 0.5	Difference of Ar = 0.4 to Ar = 0.5	Ar = 0.6	Difference of Ar = 0.5 to Ar = 0.6
0.001	1	− 7.6821	− 6.9470	0.7350	− 6.4118	0.5352
0.001	2	− 6.9680	− 6.0941	0.8739	− 5.5612	0.5329
0.001	3	− 6.3687	− 5.4409	0.9278	− 4.9389	0.5020
0.001	4	− 5.8392	− 4.9799	0.8594	− 4.5727	0.4072
0.01	1	− 7.7217	− 6.9968	0.7249	− 6.4610	0.5358
0.01	2	− 7.0273	− 6.1628	0.8645	− 5.6348	0.5280
0.01	3	− 6.4488	− 5.5254	0.9233	− 5.0152	0.5102
0.01	4	− 5.9335	− 5.0468	0.8867	− 4.6217	0.4252
0.1	1	− 8.2845	− 7.6547	0.6298	− 7.2081	0.4466
0.1	2	− 7.7856	− 7.0756	0.7101	− 6.5408	0.5348
0.1	3	− 7.4304	− 6.6332	0.7972	− 6.1176	0.5156
0.1	4	− 7.1222	− 6.2722	0.8499	− 5.7506	0.5217
0.9	1	− 8.9670	− 8.4637	0.5033	− 8.0447	0.4190
0.9	2	− 8.9396	− 8.4468	0.4928	− 8.0205	0.4263
0.9	3	− 8.9068	− 8.4208	0.4860	− 7.9837	0.4371
0.9	4	− 8.8719	− 8.3856	0.4863	− 7.9278	0.4578

### The average skin friction coefficient

**Figure 10:**
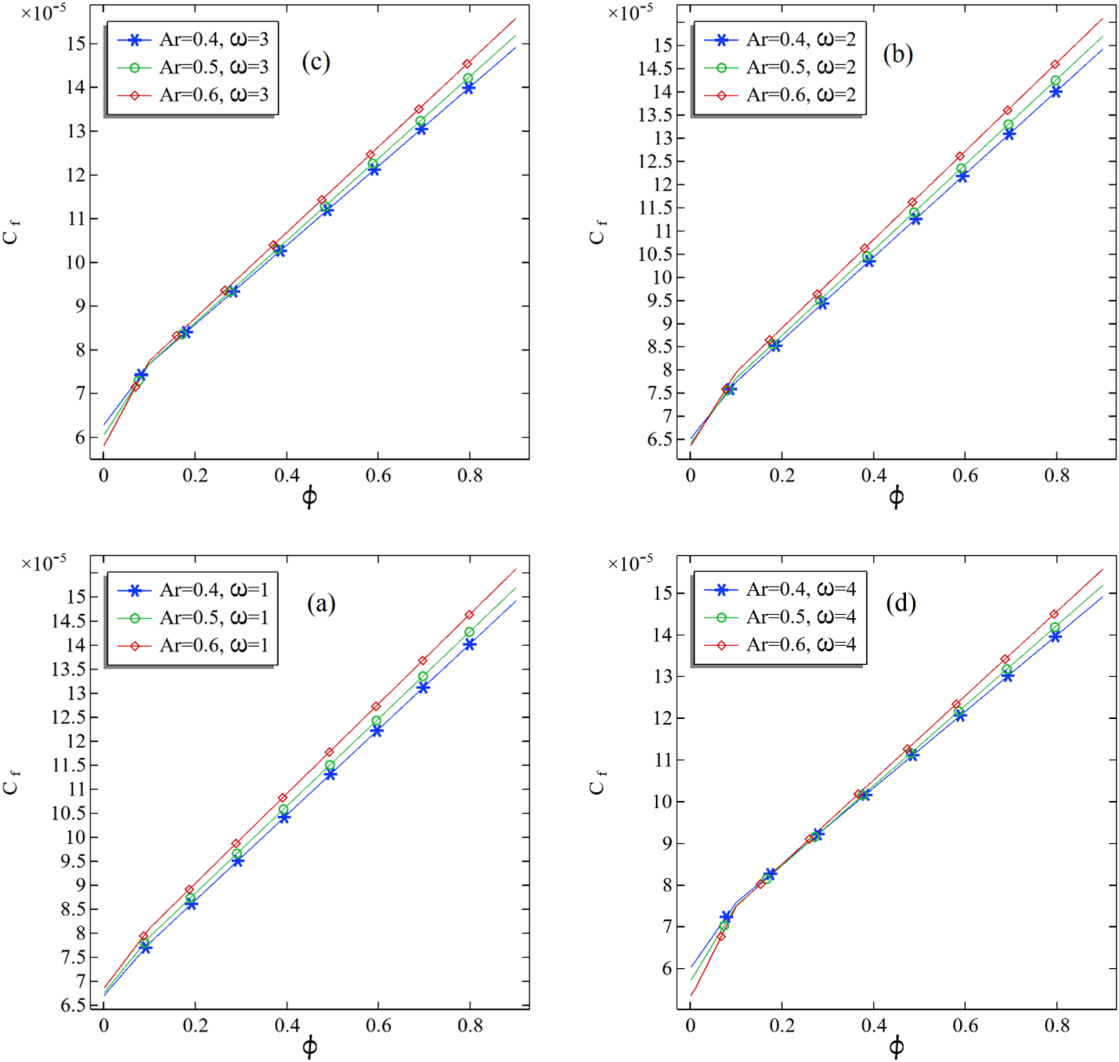
(**a**–**d**): The average skin friction coefficient at the middle of the annular with increasing volume fraction.

The skin friction due to the fluid flow is directly related to the pressure gradient and is inversely associated with flow speed. Due to the increase in nanofluids' volume fraction, the nanofluids' density is decreasing. This may increase skin friction. It is well known that skin friction will increase the heat transfer rate in the domain. The definition of skin friction coefficient is a non-dimensional number and is the ratio between shear stress to dynamic pressure. In this part, we present the impacts of volume fraction and the rotation of the inner cylinder on the average skin friction coefficient by fixing the aspect ratio. In Fig. 10a–d, we can see that the average skin friction increases linearly with boosting the volume fraction for a fixed aspect ratio. On increasing the aspect ratio, the average skin friction increases for the inner cylinder's fixed rotation. We found negligible decrement in the average skin friction coefficient when comparing these four graphs in Fig. 10a–d.

**Figure 11:**
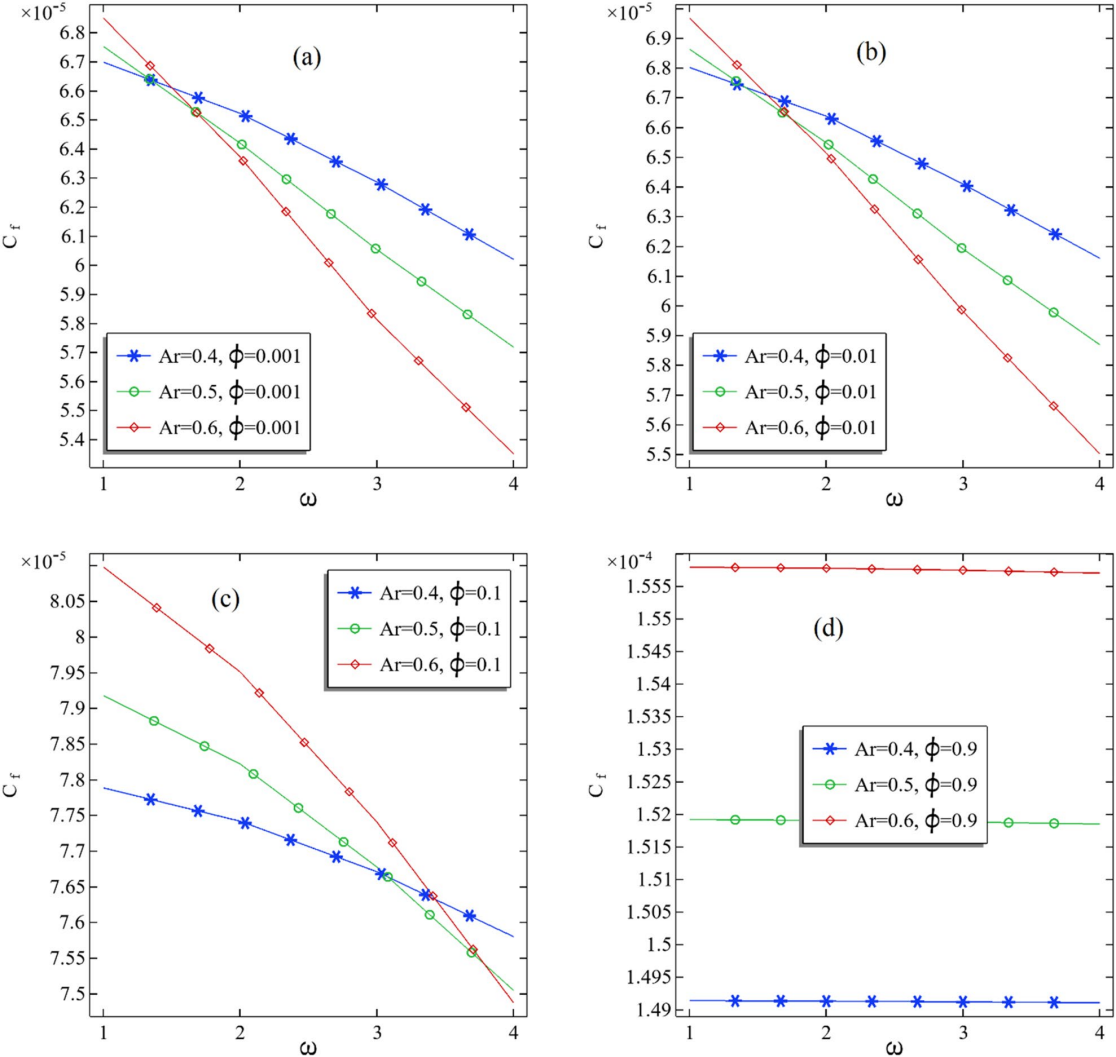
(**a**–**d**): The average skin friction coefficient at the middle of the annular with increasing rotational speed of the inner cylinder.

In Fig. 11a–d, the numerical results are presented for the average skin friction against the increase in rotational speed of the inner cylindrical. A non-functional pattern due to changing aspect ratio in the average skin friction is founded in increasing rotational speed in Fig. 11a–c. Also, it can be seen in Fig. 11d that the average skin friction is increased by increasing the aspect ratio.

In Table 5, we have presented all values of average skin friction. Also, we have added the column that illustrates the difference in average friction when the aspect ratio changes from one to another. It can be seen from Table 5 that the change of average skin friction is altered by the increasing rotational speed of the inner cylinder. This shows a non-functional pattern of skin fraction against the rotational speed. However, switching from one aspect ratio to another, the changing of average skin sometimes increased and sometimes decreased, showing a non-functionality of skin friction over aspect ratio.

**Table 5:** All the values of the average skin friction coefficient in the middle of the annular.

$\phi$	$\omega$	Ar = 0.4	Ar = 0.5	% Improved from Ar = 0.4 to Ar = 0.5	Ar = 0.6	% Improved from Ar = 0.5 to Ar = 0.6
0.001	1	6.70E-05	6.75E-05	8.09E-01	6.85E-05	1.44E + 00
0.001	2	6.52E-05	6.42E-05	− 1.57E + 00	6.37E-05	– 7.43E-01
0.001	3	6.29E-05	6.05E-05	− 3.70E + 00	5.81E-05	– 3.99E + 00
0.001	4	6.02E-05	5.72E-05	− 5.02E + 00	5.35E-05	– 6.42E + 00
0.01	1	6.80E-05	6.86E-05	9.01E− 01	6.97E-05	1.52E + 00
0.01	2	6.64E-05	6.55E-05	− 1.35E + 00	6.51E-05	– 5.31E-01
0.01	3	6.41E-05	6.19E-05	− 3.41E + 00	5.98E-05	– 3.39E + 00
0.01	4	6.16E-05	5.87E-05	− 4.72E + 00	5.50E-05	– 6.25E + 00
0.1	1	7.79E-05	7.92E-05	1.66E + 00	8.10E-05	2.27E + 00
0.1	2	7.74E-05	7.82E-05	1.03E + 00	7.95E-05	1.65E + 00
0.1	3	7.67E-05	7.68E-05	8.64E-02	7.74E-05	8.22E-01
0.1	4	7.58E-05	7.50E-05	− 9.93E-01	7.49E-05	– 2.30E-01
0.9	1	1.49E-04	1.52E-04	1.86E + 00	1.56E-04	2.55E + 00
0.9	2	1.49E-04	1.52E-04	1.86E + 00	1.56E-04	2.55E + 00
0.9	3	1.49E-04	1.52E-04	1.85E + 00	1.56E-04	2.55E + 00
0.9	4	1.49E-04	1.52E-04	1.84E + 00	1.56E-04	2.54E + 00

## Conclusion

In this article, we have examined the water aluminum oxide nanofluid through 3-dimensional annular geometry, which consisted of two concentric circular cylinders. The inner cylinder was capable move in the tangential direction with the speed of 1–4 m/s, whereas a slip boundary condition was imposed on the surface of the outer cylinder to ignore the viscous effects. A cold and hot temperature was imposed on the inner and outer cylinders. The nanofluid volume fraction was tested as 0.001, 0.01, 0.1, and 0.9, along with aspect ratios 0.4, 0.5, and 0.6. The three-dimensional incompressible Navier Stokes equation and heat energy equation were considered to obtain the simulation on COMSOL Multiphysics 5.6. A mesh independence test was employed to obtain the numerical solution for the average pressure at the outer surface of the channel. The results were compared for the average Nusselt number along the z-direction with those available by the correlations. The results were presented using graphs and tables for the average Nusselt number, percentage change in the temperature, and average skin friction in the middle of the channel. We conclude with the following points.

- The average Nusselt number at the middle plane parallel to XY-plane for a fixed rotational speed of inner cylinder is increasing linearly with the volume fraction when $\phi < 0.2$ and decreasing when $\phi > 0.2$. The average Nusselt number is improving with the increase in aspect ratio. The average Nusselt number is also improving with the increase in rotational speed by fixing the aspect ratio and the nanofluid volume fraction.
- It was determined that the largest improvement in the average Nusselt number in switching from the aspect ratio of 0.4 to 0.5 exists when $$\phi = 0.001$$$$\omega = 4$$. Similarly, the smallest decrement in the Nusselt number was observed when $$\phi = 0.9$$ and $$\omega = 1$$
- The average temperature percentage decreases with the increased volume fraction for all the aspect ratios for a fixed inner cylinder rotation. Whereas for the fixed volume fraction, the average percentage temperature along the middle of the channel increases with the increase in rotation of the inner cylinder. In both cases, the average percentage temperature improves with the increase in aspect ratio.
- It was determined that the maximum difference in the average percentage change occurs in the case $\phi = 0.001$ and $\omega = 3$ when the aspect ratio between the cylinders changes from 0.4 to 0.5. Also, when switching from 0.5 to 0.6, the maximum average percentage change can be seen in $\phi = 0.01$ $\omega = 3$ and.
- When the rotations are fixed, the average skin friction at the middle of the channel increases with an increase in the nanofluid volume. With increased rotation, the skin friction decreases significantly by about 10–23% for the particular case. But the skin friction shows a non-functionality due to the increased aspect ratio.

## Data Availability

The datasets used and/or analysed during the current study available from the corresponding author on reasonable request.
